# New liver window width in detecting hepatocellular carcinoma on dynamic contrast-enhanced computed tomography with deep learning reconstruction

**DOI:** 10.1007/s12194-024-00817-7

**Published:** 2024-06-05

**Authors:** Naomasa Okimoto, Koichiro Yasaka, Shinichi Cho, Saori Koshino, Jun Kanzawa, Yusuke Asari, Nana Fujita, Takatoshi Kubo, Yuichi Suzuki, Osamu Abe

**Affiliations:** Department of Radiology, Graduate School of Medicine, The University of Tokyo, 7-3-1 Hongo, Bunkyo-ku, Tokyo, 113-8655 Japan

**Keywords:** Hepatocellular carcinoma, CT, Deep learning reconstruction, Window width

## Abstract

Changing a window width (WW) alters appearance of noise and contrast of CT images. The aim of this study was to investigate the impact of adjusted WW for deep learning reconstruction (DLR) in detecting hepatocellular carcinomas (HCCs) on CT with DLR. This retrospective study included thirty-five patients who underwent abdominal dynamic contrast-enhanced CT. DLR was used to reconstruct arterial, portal, and delayed phase images. The investigation of the optimal WW involved two blinded readers. Then, five other blinded readers independently read the image sets for detection of HCCs and evaluation of image quality with optimal or conventional liver WW. The optimal WW for detection of HCC was 119 (rounded to 120 in the subsequent analyses) Hounsfield unit (HU), which was the average of adjusted WW in the arterial, portal, and delayed phases. The average figures of merit for the readers for the jackknife alternative free-response receiver operating characteristic analysis to detect HCC were 0.809 (reader 1/2/3/4/5, 0.765/0.798/0.892/0.764/0.827) in the optimal WW (120 HU) and 0.765 (reader 1/2/3/4/5, 0.707/0.769/0.838/0.720/0.791) in the conventional WW (150 HU), and statistically significant difference was observed between them (*p* < 0.001). Image quality in the optimal WW was superior to those in the conventional WW, and significant difference was seen for some readers (*p* < 0.041). The optimal WW for detection of HCC was narrower than conventional WW on dynamic contrast-enhanced CT with DLR. Compared with the conventional liver WW, optimal liver WW significantly improved detection performance of HCC.

## Introduction

In 2020, primary liver cancer is the sixth most common cancer and caused cancer-related deaths worldwide [1]. Hepatocellular carcinoma (HCC) accounts for 75–85% of primary liver cancer, and hepatitis B virus, hepatitis C virus, and alcohol abuse are the main risk factors of HCC [2]. There are various treatment plans for HCC: surgery, local ablation therapy, liver transplantation, transcatheter arterial chemoembolization, and systemic therapies [3]. According to the Barcelona Clinic Liver Cancer guideline [4], the size and number of HCCs play important roles in determining the treatment strategy. As a result, the accurate imaging diagnosis of HCC is essential in choosing the appropriate treatment options. In addition, unlike other tumors, most HCCs can be identified using images, and treatment can start without invasive biopsy or surgery [5]. The American Association for the Study of Liver Diseases recommends using CT or MRI for detection of HCC [6]. CT is more readily accessible and takes less time to perform than MRI. Meanwhile, it has been found that MRI is more accurate than CT at detecting HCC [7]. Image noise associated with CT image would be one of the reasons for this relatively lower HCC-detection performance because the liver mass detection performance is known to be inversely correlated with image noise [8].

In the imaging diagnosis with CT, window setting (WS) (i.e., window width [WW] and window level [WL]) is generally adjusted depending on the type of lesions to detect [9–11]. Broader WW is commonly used to detect high contrast lesions, such as lung nodules [10]. However, narrower WW is preferred in detecting low contrast lesions, such as liver masses and brain stroke [9, 11, 12]. In general, while narrower WW shows higher detection sensitivity [13], too narrow WW sometimes makes noise more prominent and generates pseudolesions [13, 14]. Based on a previous report published in 1999 [9], WW, and WL are commonly set at 150, and 50–100 Hounsfield unit (HU) in the liver WS, respectively [9, 12].

In the field of radiology, deep learning is becoming increasingly popular [15]. Previous studies have reported that deep learning assists not only imaging diagnosis [16] but also image processing [17, 18]. Deep learning reconstruction (DLR) is such an algorithm. In comparison with conventional reconstruction algorithms, DLR lowers image noise and improves image quality [17–20]. We therefore hypothesized that WW can be narrowed than the conventional setting for DLR images and that this can improve the detection performance of HCC on DLR.

This study aimed to investigate optimal WS with DLR to detect HCCs and compare HCC detection and image quality between the optimal liver WW and conventional liver WW.

## Materials and methods

This retrospective study was approved by our Institutional Review Board, which waived the requirement for obtaining written informed consent.

### Patients

This study included all consecutive patients who underwent abdominal dynamic contrast-enhanced CT scan to detect HCC. There was an overlap in patients between the current study and a previous study [20]. However, the theme of the current study (impact of window setting on HCC detection) was different from the previous study (impact of reconstruction algorithm on HCC detection).

Patients who underwent CT from October 2021 to March 2022 with one or more HCCs were assorted to the HCC group. Patients who had four or more HCCs were excluded because these patients are not subject to local therapies, according to the BCLC guideline [4]. There were 26 patients with 42 HCCs (14 patients with 1 lesion, 8 patients with 2 lesions, and 4 patients with 3 lesions) included. The size of the lesions was as follows: < 10 mm (15 lesions), 10–20 mm (15 lesions), and ≥ 20 mm (12 lesions). Two radiologists (X and Y with experience of 5 and 12 years in diagnostic radiology, respectively) created the standard for the diagnosis of HCC, referencing the following: histopathology diagnosis (8 lesions), follow-up examinations including MRI within 6 months (22 lesions), new or increasing in size with CT examinations within 6 months (11 lesions), and a single CT examination (i.e., index test) (1 lesion).

Nine patients without HCC on abdominal dynamic contrast-enhanced CT in February and March 2022 were randomly selected and were included to the non-HCC group. The absence of HCC was established based on the following: histopathology diagnosis who underwent liver transplantation (1 patient) and follow-up CT examinations (interval between the evaluated CT and the follow-up CT; more than 12 months [2 patients], more than 8 months [5 patients], and more than 5 months [1 patient]).

**Fig. 1:**
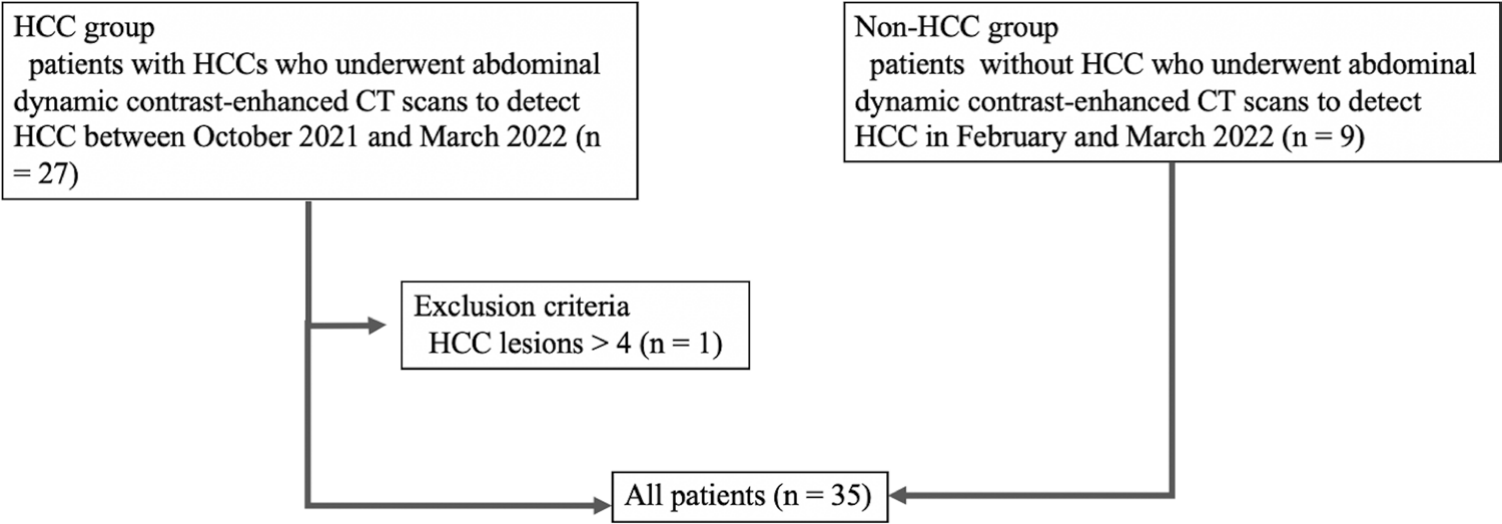
Flowchart of patient inclusion process. *HCC* hepatocellular carcinoma

Thirty-five patients (26 patients in the HCC group and 9 patients in the non-HCC group) were assigned to the final analyses. No significant difference was observed in age, sex, hepatitis B viral status, and hepatitis C viral status between the HCC and non-HCC groups (Table 1). Figure 1 shows the patient inclusion process.

**Table 1:** Demographic and clinical characteristics in the HCC and non-HCC groups

	HCC group (*n* = 26)	Non-HCC group (*n* = 9)	*p* value
Age (years: mean ± standard deviation)	73.0 ± 12.3	67.0 ± 12.1	0.180^a^
Sex (male, female)	19, 7	6, 3	0.694^b^
Hepatitis B virus (positive, negative)	5, 21	1, 8	1.000^b^
Hepatitis C virus (positive, negative)	10, 16	2, 7	0.450^b^

*HCC* hepatocellular carcinoma

^a^Mann–Whitney *U* test

^b^Fisher’s exact test

### CT imaging

A multidetector row CT (Aquilion ONE; Canon Medical Systems, Otawara, Japan) was used for all the patients. The following CT scanning parameters were used in their study: tube voltage, 120 kVp; helical pitch, 0.8125:1; gantry rotation time, 0.5 s; and tube current, automatic tube current modulation with standard deviation (SD) set at 13.0. Based on body weight, the concentration and dose of the contrast media were determined as follows: for those weighing < 50 kg, 300 mgI/mL and body weight × 2 mL, respectively; for those weighing between 50 and 60 kg, 350 mgI/mL and 100 mL, respectively; and for those weighing > 60 kg, 370 mgI/mL and 100 mL, respectively. Contrast media was injected via the antecubital peripheral vein in 30 s. The arterial, portal, and delayed phase images were scanned with the following delays: arterial phase, using a bolus tracking system (threshold attenuation of 200 HU in the descending aorta at the level of the diaphragm; portal phase, 40 s after arterial phase; and delayed phase, 180 s after the beginning of contrast agent injection). Images were reconstructed with DLR from the source data (AiCE body sharp standard, Canon Medical Systems). The following image reconstruction parameters were used: field of view, 35–40 cm (adjusted to body size), and slice thickness/interval, 3/3 mm.

CT images were anonymized and exported from the picture archiving and communication system in Digital Imaging and Communications in Medicine format.

### Investigation of optimal window setting

All the 35 image sets were randomly ordered. To investigate the optimal WW and WL, two blinded radiologists (A and B with 12 and 9 years of imaging experience, respectively) independently evaluated image sets on OsiriX (https://www.osirix-viewer.com/). They were instructed to manually modify the WW and WL in the arterial, portal, and delayed phase images to detect HCC as clearly as possible. In this investigation, WW and WL values were not displayed on the monitor (i.e., the two radiologists modified the WW and WL without knowing the WW and WL values). The determined WW and WL settings for each patient were preserved automatically. After the completion of investigation for all the patients, the preserved WW and WL settings were reviewed, and WW and WL values were recorded. Visual evaluation described in this subsection and the following two subsections were performed on a single color monitor (DELL U2718Q; 3840 × 2160, 60 Hz, 350 cd/m^2^). Brightness and contrast were set at 30 and 50%, respectively.

### HCC-detection test

Five other radiologists (readers 1, 2, 3, 4, and 5, with 6, 5, 4, 1, and 1 years of experience in diagnostic radiology, respectively) were involved in HCC-detection test under two different WW. WW120 was the optimal liver WW (WW = 120 HU) based on the previous subsection’s result, which was the rounded value for the average of adjusted WW by the radiologists in the arterial, portal, and delayed phases. For WW150, the conventional liver WW (WW = 150 HU) was adopted based on a previous report.^9^ The five readers identified HCCs and scored diagnostic confidence (4, definitely present; 3, probably present; 2, possibly present but uncertain; 1, not present) on Image J (https://imagej.nih.gov/ij/index.html). In scoring the diagnostic confidence, several image features such as early enhancement, delayed washout, size, enhancing capsule, etc. were taken into consideration comprehensively. The WW and patient information were concealed from them. They were not informed of the purpose of the study either. All the image sets (= 35 × 2) were randomly ordered. In this section, WW was fixed. However, the readers were allowed to modify WL because CT attenuation of the normal liver parenchyma can be variable based on the patient’s background liver disease (e.g., fatty liver, iron deposition, etc.) and on CT scan phase.

### Image-quality assessment

After the HCC-detection test, readers 1–5 were asked to evaluate image quality on Image J. They independently evaluated all the images in terms of the following using 5-point scale (5, clear depiction; 4, clearer than standard; 3, standard; 2, blurred than standard; and 1, unrecognizable): depiction of arterial phase hyper enhancement (APHE) and depiction of washout of HCC. In this part, only images in HCC group were included. All image sets (including both WW120 and WW150) were randomized before the evaluation. As for the WL, based on the previous subsection’s result and considering that the window level of 88 HU was suggested in a previous report [12], 90 HU was adopted as default WL for both the WW 120 and WW150. The five readers were also blinded to the WS.

### Statistical analysis

Statistical analyses were performed using EZR version 4.0.0 (https://www.jichi.ac.jp/saitama-sct/SaitamaHP.files/statmed.html) [21], which is a graphical user interface of R version 4.2.0 (https://www.r-project.org/) (R Foundation for Statistical Computing, Vienna, Austria).

The Mann–Whitney *U* test and the Fisher’s exact test were used to compare the demographic and clinical characteristics between the HCC and non-HCC groups. To evaluate the diagnostic performance for detecting HCCs with the diagnostic confidence score, jackknife alternative free-response receiver operating characteristic analysis was performed with R package of “RJafroc,” and the figure of merit (FOM), which is an analog to the area under the curve in the conventional receiver operating characteristic analysis, was obtained. To analyze the sensitivity for the detection test, diagnostic confidence scores of 2 or more were considered as positive for the presence of lesions. The sensitivities were compared between WW120 and WW150 with McNemar’s test. The Wilcoxon signed-rank test was used for the comparison of image-quality scores between WW120 and WW150. For these comparisons, *p* < 0.05 was considered to indicate statistical significance.

## Results

### Investigation of optimal window setting

The detailed results of optimal WS are shown in Table 2. The mean optimal WS (WW, WL) in all phases (mean ± SD) were (119.2 ± 28.0 and 87.5 ± 14.2 HU) by radiologist A and (118.5 ± 12.5 and 80.2 ± 15.4 HU) by radiologist B. For ease in future daily clinical practice use, values were rounded, and WW of 120 HU was adopted for WW120. As for the WL, considering that the window level of 88 HU was suggested in a previous report [12] and that of 80.2 or 87.5 HU were indicated to be optimal from our study indicated, 90 HU was adopted as default WL for both the WW 120 and WW150. However, as described in Materials and methods section, the readers were allowed to modify WL.

**Table 2:** Results for the investigation of optimal window setting

	Radiologist A		Radiologist B	
	WW (HU)	WL (HU)	WW (HU)	WL (HU)
Arterial phase	112.9 ± 21.6	84.3 ± 10.0	112.7 ± 13.1	67.5 ± 10.1
Portal phase	133.4 ± 32.2	98.1 ± 13.9	123.4 ± 11.5	92.9 ± 13.4
Delayed phase	111.2 ± 24.0	80.1 ± 12.0	119.5 ± 10.7	80.3 ± 10.6
All	119.2 ± 28.0	87.5 ± 14.2	118.5 ± 12.5	80.2 ± 15.4

Mean ± standard deviation (Hounsfield unit) are shown

*HU* Hounsfield unit, *WL* window level, *WW* window width

### HCC-detection test

**Fig. 2:**
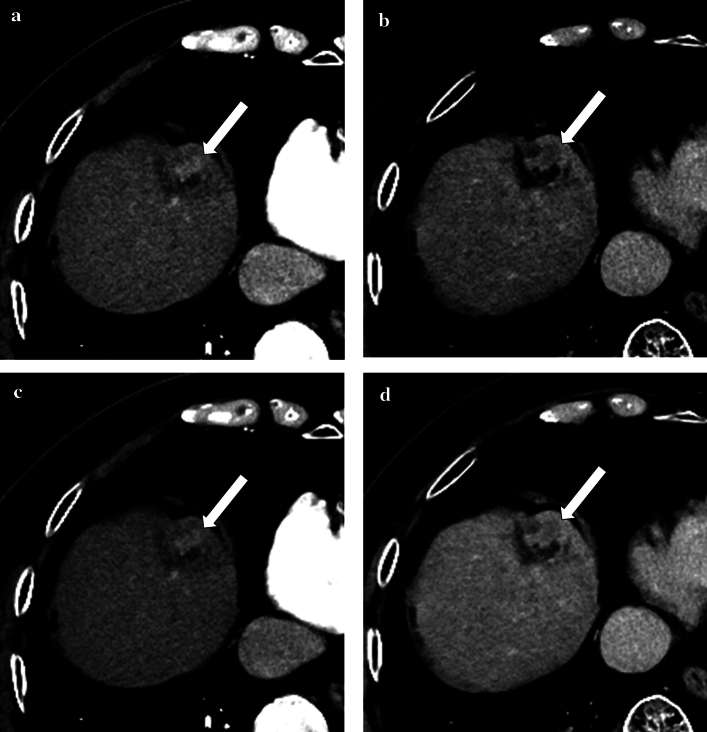
Axial dynamic contrast-enhanced CT images of a 85-year-old man with hepatocellular carcinoma (HCC) (arrows), which were reconstructed with deep learning reconstruction. Optimal window setting (window width of 120 HU) (**a**, **b**) and conventional liver window setting (window width of 150 HU) (**c**, **d**), in the arterial phase (**a**, **c**) and delayed phase (**b**, **d**) images are shown. Readers 2, 4, and 5 identified the HCC with the diagnostic confidence score of 3, 2, and 2 for the optimal window width. All readers missed this lesion in the conventional window width

**Fig. 3:**
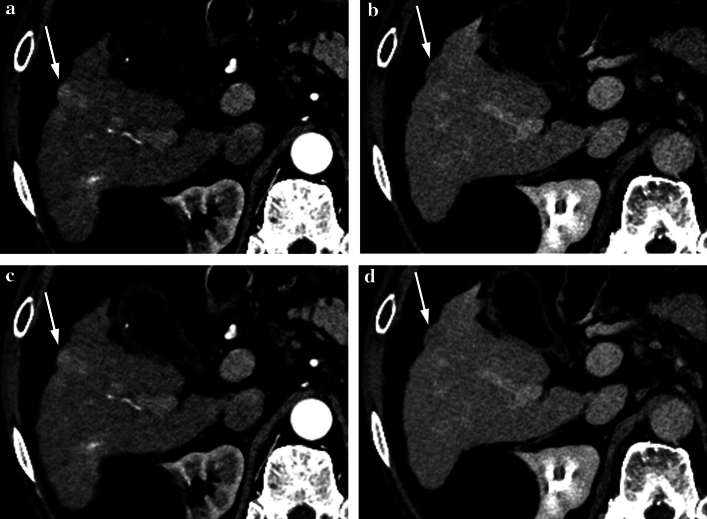
Axial dynamic contrast-enhanced CT images of a 65-year-old woman with hepatocellular carcinoma (HCC) (arrows), which were reconstructed with deep learning reconstruction. Optimal liver window setting (window width of 120 HU) (**a**, **b**) and conventional liver window setting (window width of 150 HU) (**c**, **d**), in the arterial phase (**a**, **c**) and delayed phase (**b**, **d**) images are shown. Reader 1, 2, 3, 4, and 5 detected this HCC with the diagnostic confidence score of 3, 3, 3, 3, and 2, respectively for the optimal window width. Reader 1 and 3 detected this lesion with the confidence score of 3 and 2, respectively for conventional window width

The results of the HCC-detection test are summarized in Table 3. Figures 2 and 3 represent CT images with WW120 and WW150.

**Table 3:** Results for HCC-detection test

	Reader 1	Reader 2	Reader 3	Reader 4	Reader 5	Mean (95% CI)
WW120	0.765	0.798	0.892	0.764	0.827	0.809 (0.730–0.888)
WW150	0.707	0.769	0.838	0.720	0.791	0.765 (0.674–0.856)
*p* value						< 0.001*

Figures of merit based on reader’s diagnostic confidence score with jackknife alternative free-response operating characteristic analysis are shown

*HCC* hepatocellular carcinoma, *WW* window width

^*^Statistically significant difference

**Fig. 4:**
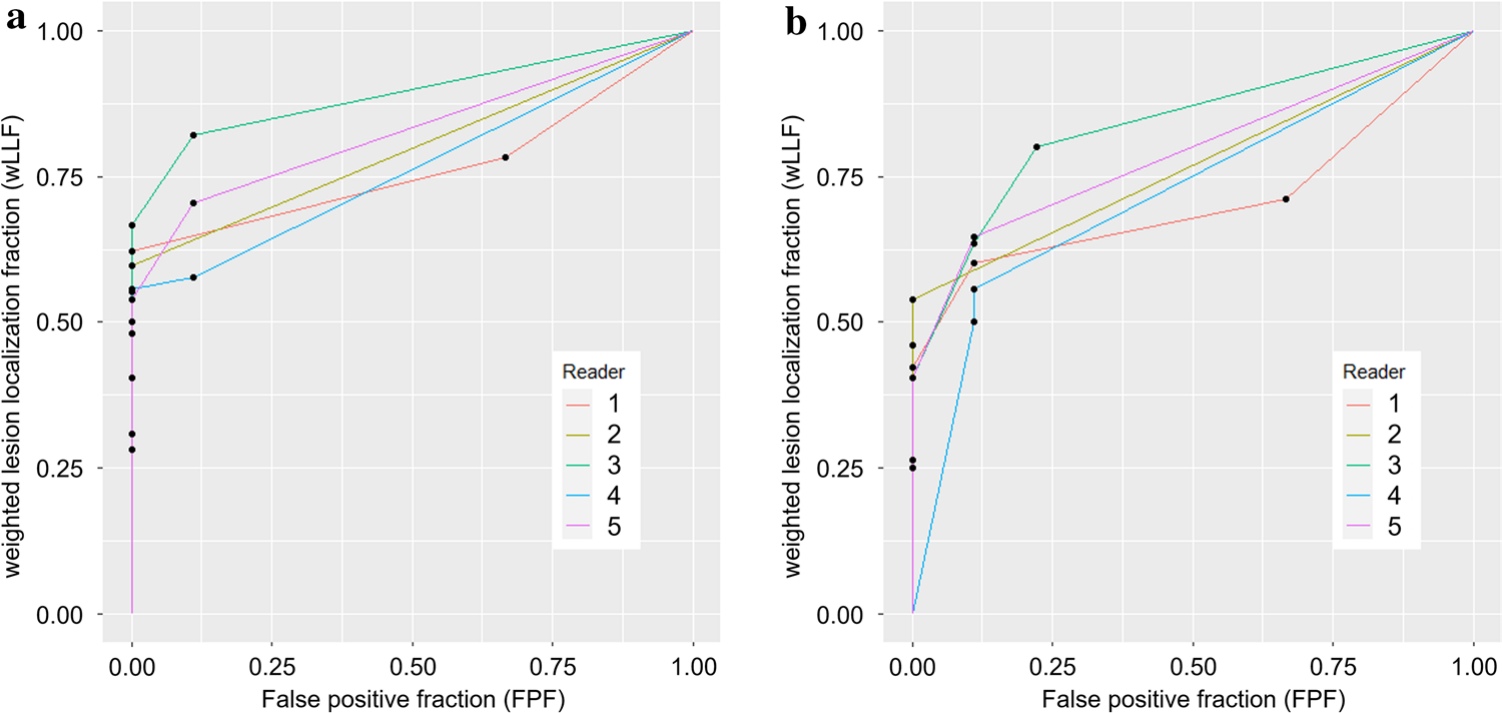
Free-response receiver operating characteristic curves for the detection of hepatocellular carcinoma in the optimal window width (120 Hounsfield unit) (**a**) and in the conventional window width (150 Hounsfield unit) (**b**). (Chakraborty DP, Zhai X. On the meaning of the weighted alternative free-response operating characteristic figure of merit. Med Phys. 2016;43(5):2548–57)

The FOM in the detection performance of HCCs averaged for the five readers were 0.809 (reader 1, 0.765; reader 2, 0.798; reader 3, 0.892; reader 4, 0.764; and reader 5, 0.827) in WW120 and 0.765 (reader 1, 0.707; reader 2, 0.769; reader 3, 0.838; reader 4, 0.720; and reader 5, 0.791) in WW150 (Fig. 4). For the detection of HCCs, the five readers performed significantly better with WW120 than with WW150 (*p* < 0.001).

The detection sensitivity of HCCs were 76, 57, 76, 52, and 67% for readers 1, 2, 3, 4, and 5, respectively, for WW120. Even though there was no statistically significant difference (reader 1, *p* = 0.248; reader 2, *p* = 0.450; reader 3, *p* = 0.617; reader 4, *p* = 1.000; and reader 5, *p* = 0.752), these values tended to be higher than those for WW150 (reader 1, 71%; reader 2, 50%; reader 3 67%; reader 4; 55%; and reader 5, 62%) except for reader 4. The numbers of false positive results were 7, 1, 3, 7, and 2 for readers 1, 2, 3, 4, and 5, respectively, for WW120 and 9, 4, 3, 4, and 2 for readers 1, 2, 3, 4, and 5, respectively, for WW150.

### Image-quality assessment

The results of the qualitative image analyses are summarized in Table 4. For readers 1, 2, 4, and 5, WW120 was significantly superior to WW150 in terms of depiction of APHE and washout of HCC (*p* < 0.041). For reader 3, though there was no significant difference between WW120 and WW150 (*p* = 0.066 for APHE), the depictions in WW120 tended to be superior to those in WW150. For reader 1, WW120 was significantly superior to WW150 in terms of depiction of washout of HCC (p < 0.001). For the other readers, though there was no significant difference between WW120 and WW150, the depiction of washout in WW120 tended to be superior to those in WW150.

**Table 4:** Results for image-quality analyses

Imaging feature	Reader	WW120	WW150	Comparison (*p *value)
Depiction of features of HCC (score 5/4/3/2/1)				
APHE	1	0/1/32/9/0	0/3/9/30/0	< 0.001*
	2	11/14/17/0/0	6/13/22/1/0	0.001*
	3	0/3/39/0/0	0/2/35/5/0	0.066
	4	0/10/32/0/0	0/4/38/0/0	0.041*
	5	2/22/17/1/0	1/11/30/0/0	0.008*
	Pooled	13/50/137/10/0	7/33/134/36/0	< 0.001*
Washout	1	0/5/22/15/0	0/0/15/27/0	< 0.001*
	2	6/22/14/0/0	2/11/29/0/0	0.088
	3	0/5/34/3/0	0/7/25/9/1	0.243
	4	1/17/19/5/0	2/7/33/0/0	0.519
	5	1/15/26/0/0	1/8/32/1/0	0.116
	Pooled	8/64/115/23/0	5/33/134/37/1	< 0.001*

Numbers of HCCs for each score are shown. Comparisons were performed using the Wilcoxon signed-rank test

*APHE* arterial phase hyper enhancement, *HCC*, hepatocellular carcinoma, *WW* window width

^*^Statistically significant difference

## Discussion

This study showed that 118.5–119.2 HU and 80.2–87.5 HU was the optimal WW and WL, respectively, to detect HCCs in abdominal dynamic contrast-enhanced CT with DLR. In comparison to conventional liver WW of 150 HU, optimal liver WW of 120 HU significantly improved HCC-detection performance (FOM of 0.809 for WW120 and 0.765 for WW150, *p* < 0.001).

There are various HCC-treatment options. The stage of HCC determined by imaging plays an important role in deciding treatment strategy. The Barcelona Clinic Liver Cancer guideline in 2022 states that local treatment, such as surgery and local ablation therapy, are likely to be selected for patients with very early or early stages, and systematic therapies and drugs, such as small molecule targeted drugs, are usually used for patients with intermediate or advanced stages [22, 23]. DLR with optimal liver WS, which we propose, may help radiologists detect HCC with earlier stages. As for the selection of treatment strategies, hepatectomy or radiofrequency ablation is typically indicated for patients with Child–Pugh A or B, lacking extrahepatic metastasis or vascular invasion, and presenting with up to three HCC (≤ 3 cm). Otherwise, transcatheter arterial chemoembolization, chemotherapy, or palliative care are usually selected. Because the accurate detection of intrahepatic HCC is crucial in selecting treatment plans, our result may have the ability to improve prognosis by providing appropriate treatment strategies to patients.

The optimal liver WS resulted in increasing the conspicuity of the features of HCC, which was associated with higher diagnostic performance in detecting HCCs (FOM = 0.764–0.892) as compared to a similar previous report (FOM = 0.731) [24]. Previous studies have shown that liver WS is needed in addition to soft tissue setting in detecting liver lesions [9, 25]. One study has reported that additional liver lesions were identified with liver WS in 3.1% patients [9]. In the present study, the sensitivity in detecting HCCs were improved with optimal liver WS compared to conventional WS for most readers. On the contrary, narrow WW is known to cause noisy contrast and pseudolesions such as arterioportal shunts [13, 14]. Actually, the number of false positives in WW120 was increased compared to that in WW150 for one reader. However, in this study, the numbers of false positive findings in the optimal WS were equal to or less than those in conventional WS for the other 4 readers. This would be due to the reduced image noise associated with DLR, which had already been reported in several previous articles [18, 26]. The noise reduction with DLR keeps image conspicuity without noise distracting, even with narrow WW for most readers. It is difficult to identify the washout of small HCC on CT [27]. In the present study, 71% (30/42) of the lesions were ≤ 20 mm in size, but the depiction of washout tended to be superior in WW120 for all readers. This may have influenced the outcomes of this study.

This study has some limitations. First, the reference standard for the diagnosis of HCC was not established histopathologically for all lesions. In daily clinical practice, HCCs are usually diagnosed with images, and treatment is performed without invasive procedure. However, it is considered practically acceptable even if we could not confirm that all hepatic lesions were HCC. Second, the study only focused on HCC. It remains unclear whether optimal liver WS could match the detection of other hepatic lesions. Further studies would be necessary. Third, the evaluations of images with WW120 and WW150 were performed at once without time interval, which may be associated with a recall bias. However, because dataset of WW120 and WW150 were randomized, we believe that it would not affect the main results (i.e., WW120 was superior to WW150). Fourth, while there was a report regarding deep learning algorithm which detect HCC [28], how such algorithm performs on dynamic contrast-enhanced CT with DLR was not evaluated. Future research regarding this topic would be necessary. Fifth, we did not compare the detection performance between CT and MRI. The difference between narrower window width on CT images reconstructed with DLR and MRI needs to be assessed in future studies. Sixth, the number of patients without HCC was relatively small compared to those with HCC. However, there are several CT slices without HCC even in patients with HCCs. In setting those numbers, we referred to a previous article which assessed the observer performance of brain metastases on CT images [29]. In that study, 25 cases with brain metastases and 5 cases without brain metastases were included. In addition, to reduce the bias by readers, readers were blinded to the information regarding the numbers of patients with HCC and those without HCC. Seventh, the total number of patients included in this study was also relatively small. However, because statistically significant improvement in the main result was observed, future research including larger numbers of patients would be warranted. Eighth, while body weight is one of the important factors that is associated with the degree of noise, this was not recorded in our study. However, because the same patient data were used between WW120 and WW150, that would not have a major impact on the main result. Ninth, the qualitative evaluations were performed not on a medical monitor. However, because both WW120 and WW150 images were randomized before the evaluation, it would not affect the main result of our study. Finally, DLR nor CT device by each manufacturer does not adopt identical algorithms; therefore, the results in this study do not always apply to other DLRs or CT devices. In addition, our result would not necessarily directly applicable to CT images which were acquired with other scan or reconstruction parameters.

In conclusion, the optimal WW for detection of HCC on dynamic contrast-enhanced CT was narrower for DLR images than previous reports. Compared with the conventional liver WS (WW of 150 HU), the optimal liver WS (WW of 120 HU) significantly improved detection performance of HCC.

## Data Availability

Due to the nature of this research, patients of this study did not agree for their data to be shared publicly, so supporting data is not available.
